# Multi-Step Apparent Temperature Prediction in Broiler Houses Using a Hybrid SE-TCN–Transformer Model with Kalman Filtering

**DOI:** 10.3390/s25196124

**Published:** 2025-10-03

**Authors:** Pengshen Zheng, Wanchao Zhang, Bin Gao, Yali Ma, Changxi Chen

**Affiliations:** 1 Key Laboratory of Smart Breeding (Co-Construction by Ministry and Province), Ministry of Agriculture and Rural Affairs, Tianjin 300384, China; zhengpengshen_9206@163.com (P.Z.); cssiiyo26@163.com (W.Z.); bingao_1594@163.com (B.G.); mayali_7447@163.com (Y.M.); 2 College of Computer and Information Engineering, Tianjin Agricultural University, Tianjin 300384, China

**Keywords:** apparent temperature prediction, broiler house environment, heat stress, TCN, transformer, Kalman filter, Squeeze-and-excitation, precision livestock farming

## Abstract

In intensive broiler production, rapid environmental fluctuations can induce heat stress, adversely affecting flock welfare and productivity. Apparent temperature (AT), integrating temperature, humidity, and wind speed, provides a comprehensive thermal index, guiding predictive climate control. This study develops a multi-step AT forecasting model based on a hybrid SE-TCN–Transformer architecture enhanced with Kalman filtering. The temporal convolutional network with SE attention extracts short-term local trends, the Transformer captures long-range dependencies, and Kalman smoothing reduces prediction noise, collectively improving robustness and accuracy. The model was trained on multi-source time-series data from a commercial broiler house and evaluated for 5, 15, and 30 min horizons against LSTM, GRU, Autoformer, and Informer benchmarks. Results indicate that the proposed model achieves substantially lower prediction errors and higher determination coefficients. By combining multi-variable feature integration, local–global temporal modeling, and dynamic smoothing, the model offers a precise and reliable tool for intelligent ventilation control and heat stress management. These findings provide both scientific insight into multi-step thermal environment prediction and practical guidance for optimizing broiler welfare and production performance.

## 1. Introduction

Poultry farming is a vital sector of global agriculture, playing a key role in ensuring food security and driving economic growth [1]. This underscores the strategic importance of the poultry industry within future food supply systems. In this context, broiler production increasingly depends on a stable and controllable farming environment [2]. Due to dense plumage and lack of sweat glands, broilers are highly sensitive to heat stress, which affects behavior, physiology, and immunity, leading to reduced feed intake, slower growth, decreased feed conversion efficiency, and increased production costs [3,4,5,6].

In recent years, the transition toward large-scale and intensive poultry farming has heightened attention to microclimate regulation within poultry houses. Environmental factors including temperature, humidity, gas concentrations, and airflow distribution directly or indirectly influence flock health, growth, and immune function [7]. Maintaining optimal rearing conditions is thus essential for welfare and production efficiency. Traditional control methods, typically based on static thresholds or empirical rules, often fail to accommodate external disturbances and dynamic physiological needs, resulting in heat stress, respiratory diseases, and growth impairments [8]. Thermal comfort in poultry arises from nonlinear interactions among multiple environmental factors; for example, high humidity (>60%) impedes heat dissipation under high temperatures but facilitates heat exchange under moderate temperatures [9]. Ventilation is also crucial, as increased airflow lowers surface temperature, enhances convective and evaporative cooling, and improves thermal comfort [10,11]. Furthermore, thermal perception varies by growth stage and body region, influenced by both environmental and intrinsic physiological factors such as age [12]. Consequently, single-parameter thresholds inadequately represent the flock’s actual thermal load. A comprehensive thermal comfort index integrating temperature, humidity, and wind speed, adjusted dynamically by age, is necessary to guide precise environmental control and stress warning systems.

To date, numerous studies have proposed various heat indices and mitigation strategies to quantify and alleviate heat stress. These studies emphasize the multifactorial nature of heat stress and advocate combining environmental indices with management measures to reduce its negative impacts [13]. Experimental and modeling evidence further indicates that accurate microclimate assessment requires not only high temporal resolution but also spatial distribution information, as local airflow patterns and sensor placement can substantially influence the perception of heat load. Apparent temperature (AT) is an empirical index based on effective temperature theory [14], considering both the cooling effect of wind and the heat load change caused by humidity deviation. It has been widely applied to broiler house climate control, providing a practical basis for ventilation, heating, and cooling to ensure optimal thermal environment, thereby improving growth performance and health. Its simple structure and adjustable parameters are easy to integrate into automated ventilation systems for dynamic estimation of the thermal environment and planning of rearing strategies.

The development of IoT technologies, intelligent algorithms, and control systems has driven precision livestock farming (PLF) based on multi-source data fusion, enabling intelligent and sustainable poultry production [15,16]. Practical applications have demonstrated the effectiveness of these approaches. For instance, deep learning-based broiler counting methods significantly improve detection accuracy and speed, supporting efficient flock management and enabling smart poultry farming transitions [17]. Automated climate control systems provide economic and environmental benefits: fully automated houses reduce costs, while real-time optimization strategies minimize deviations in ventilation setpoints [18,19]. Sensor networks combined with machine learning models enable automatic regulation of multiple parameters, reducing labor dependence [20]. Innovations such as combined mechanical–natural ventilation and positive–negative pressure systems based on dynamic decoupling models optimize airflow and temperature distribution [21,22]. Integration of IoT and intelligent algorithms enables adaptive, self-optimizing ventilation, with simulation tools supporting energy assessment and welfare enhancement [23].

At the same time, physically based modelling—particularly computational fluid dynamics (CFD) and coupled heat–mass transfer simulations—has provided detailed insight into airflow patterns, temperature stratification, and pollutant transport within animal houses. CFD studies demonstrate that high-fidelity physical models can quantify localized airflow distribution and predict hotspots, offering mechanistic interpretability that can guide ventilation design and sensor layout [24,25]. However, such high-resolution physical modelling typically requires detailed geometric and boundary condition data (e.g., fan characteristics, inlet distributions, and heat and moisture transfer coefficients) and is computationally intensive, which complicates real-time or online deployment for multi-step control tasks. Therefore, while CFD and physics-based models are invaluable for design and diagnosis, their direct use in rapid, multi-step forecasting and control remains limited in practice.

Despite these advances, real-time monitoring and control systems still face inherent delays in data transmission, decision-making, and actuator response, limiting their ability to respond promptly to rapid environmental changes. Air pollution and dust interference further compromise data timeliness and integrity [26]. Therefore, systems relying solely on real-time feedback cannot anticipate environmental changes, delaying corrective actions and threatening flock health and performance. Developing predictive models capable of forecasting environmental dynamics is thus essential.

Data-driven modeling excels at capturing nonlinear patterns and has been widely applied to environmental prediction in poultry houses [27]. Early work correlated chicken behavior with environmental variables to evaluate rearing conditions using data mining techniques [28]. Integrations of energy conservation principles with structural and biological parameters yielded microclimate dynamic simulation models combining physical and data-driven methods [29]. Deep learning techniques have accurately predicted CO_2_ concentrations in layer and pig houses [30,31], and neural networks have forecasted harmful gases like CO_2_ and NH_3_ in broiler houses, advancing intelligent gas monitoring [32,33]. Hybrid models combining metaheuristic optimization algorithms and deep networks—such as dung beetle optimization with TCN-GRU—show strong robustness and accuracy in dynamic, disturbed environments like piglet barns [34], indicating broad applicability to livestock environmental modeling.

Recent advances in time-series deep learning further expand the modeling toolbox. Convolutional sequence models such as Temporal Convolutional Networks (TCNs) have demonstrated excellent performance on short-term and medium-term sequence modelling tasks due to their effective receptive field expansion via dilations [35]. Transformer-based architectures and their long-sequence variants (e.g., Informer, Autoformer, and SCINet) have been proposed to capture long-range dependencies and multi-scale patterns in general time-series forecasting problems [36,37,38,39]. These methods bring powerful representational capacity but also introduce challenges: Transformer family models often demand careful hyperparameter tuning and substantial training data to realize their full potential, and naïve application to long raw sequences can be computationally and memory intensive. Consequently, for domain-specific applications with limited data, hybrid architectures that combine convolutional inductive biases (for local patterns) and attention mechanisms (for global patterns) frequently provide a pragmatic compromise.

In summary, livestock environmental control systems are shifting from static, reactive approaches to dynamic, predictive frameworks [40]. Accurate multi-step forecasting of environmental parameters mitigates control lags and enables proactive strategy optimization, crucial for broiler farming where thermal stress profoundly affects flock comfort and productivity. However, existing studies have predominantly focused on individual parameters or specific gases, with limited attention to high-precision, multi-step forecasting models for apparent temperature—a comprehensive thermal comfort index jointly influenced by age, temperature, humidity, and wind speed. To bridge this gap, we propose a multi-step prediction framework that integrates an SE-TCN–Transformer architecture with Kalman filtering. In this design, the SE-TCN module effectively captures short-term dynamic variations, the Transformer component models long-range temporal dependencies, and the Kalman filter enhances stability by mitigating prediction noise. This hybrid approach enables accurate forecasting of apparent temperature trends over specified future horizons, thereby supporting proactive environmental control strategies such as intelligent ventilation and precision thermal regulation. Ultimately, the proposed model contributes to improving poultry welfare and production efficiency, while highlighting its broader scientific significance and practical applicability.

## 2. Materials and Methods

### 2.1. Data Collection and Preprocessing

The experiment was conducted from 27 March to 30 April 2024 at the Smart Farming Facility in Pingyuan County, Dezhou City, Shandong Province. It covered one farming cycle of 5–6 weeks, raising over 30,000 birds. Each building measures 101 m in length and 16 m in width, with an eave height of 4.2 m and ridge height of 6 m. The poultry houses comprise 7 rows of individual cages, each with 4 layers and 73 cages per layer. Each cage measures 1.25 m (L) × 1.0 m (W) × 0.45 m (H), with an overall height of 2.55 m. The houses employ a longitudinal layout and a combined positive–negative pressure ventilation system. The system includes 24 large fans with an airflow of 35,000 m^3^/h and 2 smaller fans rated at 12,000 m^3^/h. Evaporative coolers are installed, with gable wall coolers measuring 15 m (W) × 3.66 m (H) and side coolers measuring 22.8 m (W) × 2.16 m (H). In this system, fans installed at the rear of the house exhaust air, creating a slight negative pressure inside the poultry house relative to the outdoor atmospheric pressure, which drives fresh air to enter through controlled inlets and ensures effective ventilation. Stocking density varies by growth stage: 70 birds per cage in the early phase, 30 in the middle phase, and 20 in the late phase. The average initial body weight was 45 g per bird, with a standard final weight of 1050 g.

**Figure 1:**
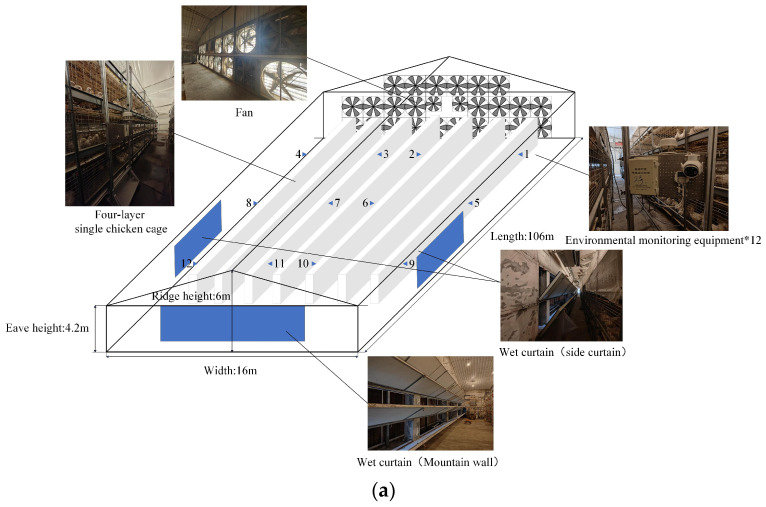

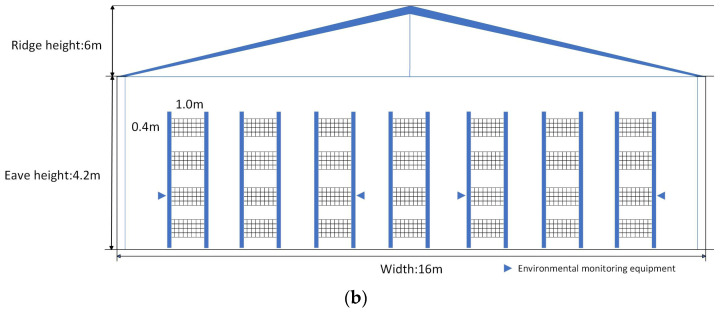
Schematic diagram of chicken coop structure and No. 1–12 monitoring equipment layout (**a**) Top view; (**b**) side view.

Figure 1 illustrates the layout of environmental monitoring equipment inside the chicken house. Horizontally, to capture environmental variations across the front, middle, and rear sections, monitoring devices are installed every 25 m along the house length at cage positions 05, 35, and 65 in columns 1, 3, 5, and 7, totaling 12 units. Vertically, since the chicken house employs a four-tier cage system, all sensors are placed at the height of the second tier to optimize coverage of vertical spatial differences.

**Figure 2:**
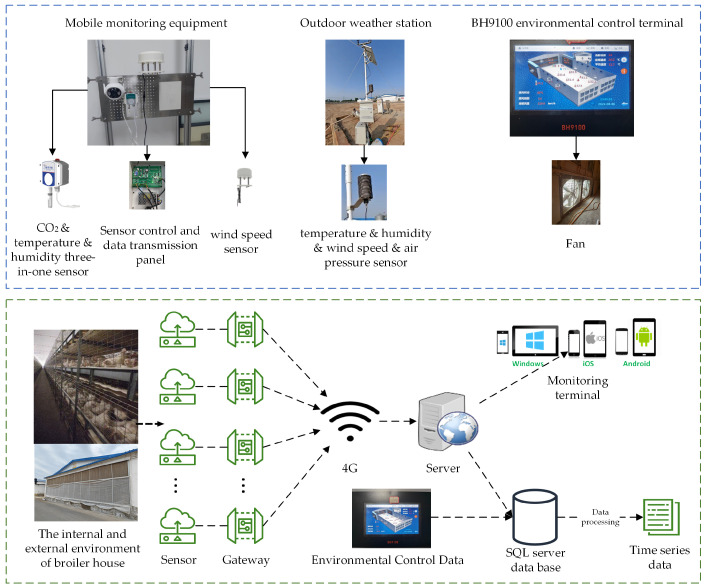
Broiler house indoor and outdoor environment experimental data collection flow chart.

Figure 2 illustrates the environmental data sensors and the data collection process inside and outside the chicken house during the experiment. Continuous real-time data were collected daily from 00:00 to 24:00 at 5 min intervals by 12 indoor monitoring units and an outdoor weather station. Monitored variables included indoor temperature ($T_{in}$), humidity (${RH}_{in}$), wind speed ($V_{in}$), CO_2_ concentration (*CO*_2_), and negative pressure ($P_{in}$), as well as outdoor temperature ($T_{out}$), humidity (${RH}_{out}$), wind speed ($V_{out}$), and atmospheric pressure ($P_{out}$). Data were transmitted to a remote server via a 4G network. Environmental control parameters—such as ventilation level (*level*), ventilation volume (*Ven*), number of operating fans (*Fan*), and target temperature ($T_{target}$) for each growth stage—were exported from the control terminal and sent to a remote database. In this experiment, the light intensity and photoperiod were kept constant according to the standard breeding management of the enterprise, so the light was not used as the input variable of the model. Sensor specifications inside and outside the chicken house are detailed in Table 1.

**Figure 3:**
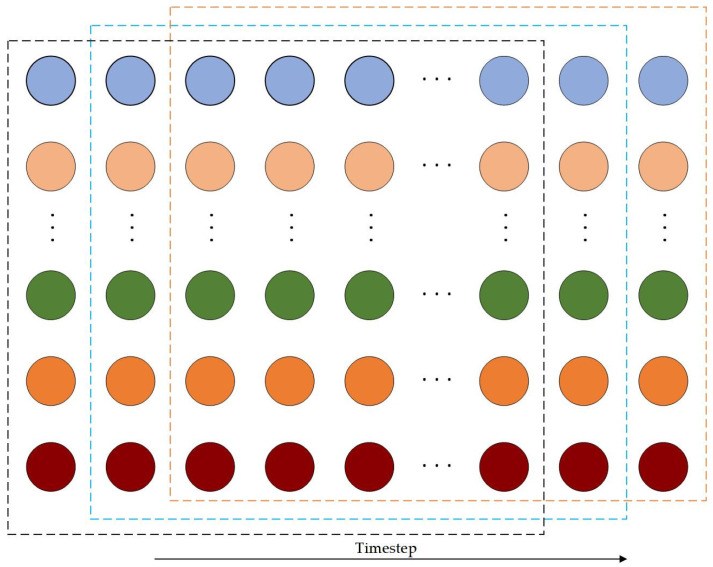
Diagram of the sliding window strategy.

The time series data collected during the experiment were used to train the apparent temperature prediction model. The dataset was split into training and test sets at an 80:20 ratio. Due to equipment instability and signal quality issues, some environmental data showed anomalies during monitoring and transmission, adversely affecting model performance. To enhance data quality and ensure efficient model training and accurate predictions, the following preprocessing steps were applied:

i. Data from all 12 indoor monitoring devices were aggregated by calculating their arithmetic mean. This provided a representative overall value for the entire facility, used to analyze real-time conditions and cyclic variations.
ii. To maintain data continuity and accuracy, missing and abnormal values were imputed based on the local linear trend within the dataset, avoiding erroneous interpolation over extended time periods.
iii. In order to evaluate the thermal environment of the henhouse, the apparent temperature (AT) was calculated based on the processed data. The apparent temperature formula is shown in Equation (1).
  (1) $$AT=T-V\times K_{v}+(RH-RH_{target})\times K_{h}$$
  where *T* is the indoor temperature (°C), *V* is the indoor wind speed (m/s), *RH* is the indoor relative humidity (%), *RH_target_* is the target humidity (%), *K_v_* is the wind chill coefficient, and *K_h_* is the wet-heat coefficient. Age-dependent coefficients are used in the apparent temperature (AT) formula to account for the varying thermal sensitivity of broilers at different growth stages. Younger chicks are more susceptible to heat or cold stress due to their underdeveloped thermoregulatory systems, while older birds have greater tolerance to environmental fluctuations. By adjusting the wind chill coefficient (*K_v_*) and wet-heat coefficient (*K_h_*) according to age, the AT calculation more accurately reflects the perceived thermal comfort of the flock, allowing for more precise environmental management and ventilation control. This approach, based on empirical studies, balances physiological relevance with practical feasibility in commercial poultry production. In this study, the wind chill coefficient and wet-heat coefficient were segmented by age, as shown in Table 2.
iv. To remove scale differences among multiple environmental features and accelerate model convergence, Min-Max-Scaler was applied to normalize all parameters, ensuring data values lie within a consistent range. The normalization formula is shown in Equation (2).
  (2) $$z=\frac{x-x_{\min}}{x_{\max}-x_{\min}}$$
  where z denotes the normalized value, x is the original data, and $x_{min}$ and $x_{max}$ represent the minimum and maximum values of the original environmental data, respectively.
v. A sliding window strategy was employed to segment the normalized time series into fixed-length input sequences and their corresponding future outputs, as illustrated in Figure 3. Each input window spanned 180 min, encompassing 36 time steps (5 min per time step), which captures short-term dynamic variations in the broiler house environment. The windows slid along the time series with a step size of one time step (5 min), generating overlapping sequences to increase the number of training samples without losing information. This approach ensures that the model can learn fine-grained temporal dependencies and repetitive patterns in the data, while minimizing the loss of transient or abrupt changes in the environment, thereby enhancing robustness and predictive performance in multi-step forecasting tasks.

**Table 2:** Changes in each parameter with age.

Age (Day)	${RH}_{target}$	Wind Chill Coefficient	Wet-Heat Coefficient
1–6	60%	8	0.12
7–13	60%	6	0.12
14–20	55%	5	0.10
21–27	55%	4	0.08
28–24	55%	3.5	0.06
35–41	55%	3	0.03
42-	55%	3	0.00

### 2.2. Model Structure

**Figure 4:**
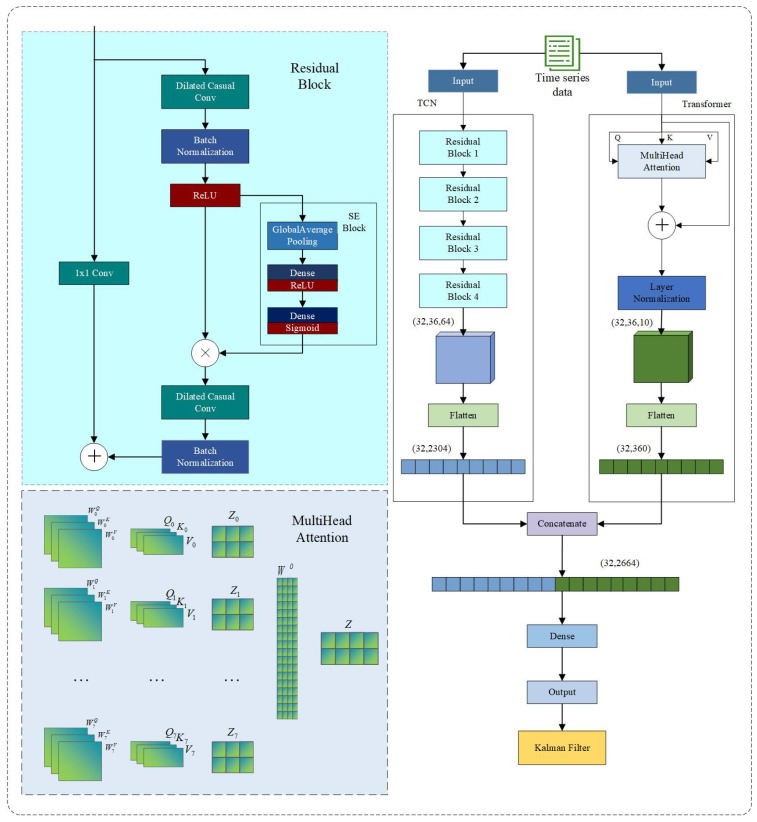
Construction of SE-TCN–Transformer–Kalman network architecture.

This study proposes a hybrid architecture combining a temporal convolutional network (TCN) and a Transformer. The TCN extracts fine-grained local temporal features, while the Transformer captures long-range global dependencies. The SE (Squeeze-and-Excitation) attention mechanism is incorporated to adaptively recalibrate channel feature weights and enhance key information representation. Finally, Kalman smoothing post-processes the model’s apparent temperature output to suppress instantaneous fluctuations, enabling high-fidelity prediction of apparent temperature dynamics. The overall framework is illustrated in Figure 4.

To fully extract multi-scale temporal features, this study designs a parallel dual-branch network consisting of a local feature extraction branch (SE-TCN) and a global dependency modeling branch (Transformer). The local branch leverages residual blocks built on Temporal Convolutional Networks (TCNs), where each block contains stacked dilated 1D convolutions that progressively expand the receptive field, allowing the network to capture short-term and mid-term temporal patterns. Each residual block integrates a Squeeze-and-Excitation (SE) module, which performs global average pooling followed by two fully connected layers with a non-linear activation, generating channel-wise attention weights to recalibrate feature importance. This mechanism enables the network to emphasize informative channels while suppressing less relevant ones, enhancing the representation of critical local fluctuations. Residual connections within each block ensure stable gradient propagation and mitigate vanishing gradients in deep TCN layers. Collectively, these components form the SE-TCN branch, effectively modeling local temporal dynamics across multiple scales.

Despite TCN’s effectiveness in local feature extraction, it has inherent limitations in capturing long-range dependencies due to its convolutional nature. To address this, the global branch employs a Transformer encoder with multi-head self-attention, which calculates attention scores across all time steps in parallel, identifying long-term dependencies and global trends. Each Transformer layer incorporates residual connections and Layer Normalization to maintain training stability and robust representation of sequential information. This branch allows the model to understand patterns that span extended temporal horizons, complementing the local branch.

The outputs of both branches are flattened and concatenated in the feature fusion layer, combining local short-term and global long-term representations into a unified sequence feature vector. This fused representation is then passed through a fully connected layer to regress future apparent temperature values, enabling precise multi-step forecasting. Finally, to enhance temporal consistency and suppress high-frequency fluctuations, a Kalman filter is applied as a post-processing step, dynamically smoothing predictions and improving overall stability and reliability in practical deployment. This dual-branch SE-TCN–Transformer architecture thus integrates multi-scale temporal extraction, attention-based feature calibration, and long-term dependency modeling, providing a robust framework for accurate and adaptive apparent temperature prediction under complex environmental fluctuations.

In the poultry house environmental control framework, the multi-step apparent temperature predictions generated by the SE-TCN–Transformer–Kalman model can be integrated into the automatic control terminal for real-time decision execution. The predicted values are continuously compared with age-specific thresholds. When forecasts exceed the upper threshold, the control terminal issues commands to increase fan speed levels or activate evaporative cooling pads; when forecasts fall below the lower threshold, it sends commands to decrease fan speed or engage auxiliary heating units. These control signals are executed through the existing ventilation and cooling infrastructure, enabling predictive adjustments several minutes before critical deviations occur. This implementation ensures that the predictive model directly drives environmental actuators, thereby compensating for inherent delays in sensor acquisition, decision processing, and equipment response.

#### 2.2.1. Temporal Convolutional Network (TCN)

**Figure 5:**
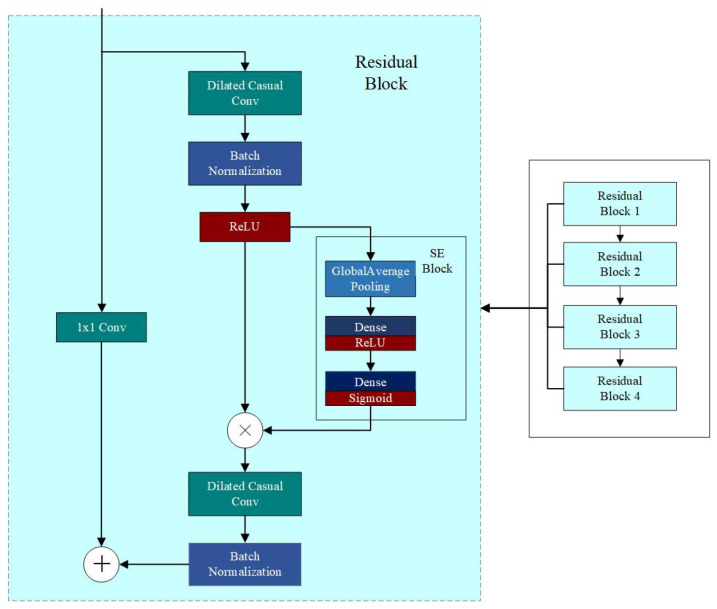
Construction of the TCN architecture.

The Temporal Convolutional Network (TCN) is a deep neural network architecture tailored for sequential data processing, extensively applied in time series prediction, speech recognition, and natural language processing. Its key components—causal convolutions, dilated convolutions, and residual connections—merge the parallel computing strengths of Convolutional Neural Networks (CNNs) with effective temporal dependency modeling. The TCN branch architecture used in this study is illustrated in Figure 5.

**Figure 6:**
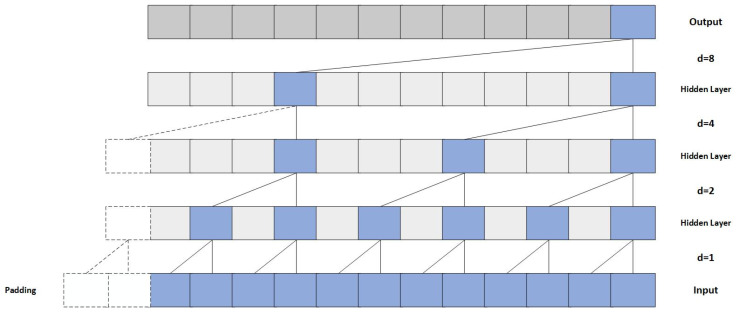
Schematic representation of causal and dilated convolution methods.

Unlike traditional recurrent neural networks (RNNs), TCNs avoid recursive mechanisms and instead apply convolution operations at each layer to extract time series features. This approach significantly enhances training efficiency and fully leverages modern hardware acceleration [41]. The causal and dilated convolutions in TCN are illustrated in Figure 6.

In TCN, causal convolution ensures that the output at each time step depends solely on the current and past inputs, strictly adhering to the causality constraints of time series. For the one-dimensional input sequence $x=[x1,x2,\ldots ,x_{t}]$, the t-th element of the convolution output is given by Equation (3).

(3) $$y_{t}=\sum_{i=0}^{K-1}\omega_{i}\cdot x_{t-i}$$

where $\omega_{i}$ denotes the i-th weight of the convolution kernel, and K represents the kernel size. The causal structure prevents future information from “leaking” into the current time step’s prediction, ensuring consistency with the temporal logic of real-world forecasting tasks.

To further expand the receptive field and capture long-term dependencies, TCN employs dilated convolutions. These convolutions insert “holes” or skip connections between kernel elements, allowing the receptive field to grow exponentially without substantially increasing the number of parameters or computational complexity. The formula is shown in Equation (4).

(4) $$y_{t}=\sum_{i=0}^{K-1}\omega_{i}\cdot x_{t-d\cdot i}$$

where d denotes the dilation rate, specifying the spacing between input elements. By stacking multiple dilated convolution layers with dilation rates d=1,2,4,8, the TCN captures multi-scale temporal features, from short-term fluctuations to medium- and long-term trends, while keeping computational costs low.

Each convolutional module in the TCN is followed by batch normalization and ReLU activation to accelerate convergence and improve nonlinear modeling. Residual connections in deep layers alleviate the vanishing gradient problem and enhance model stability and generalization. Finally, temporal features extracted through multiple dilated convolution layers and residual blocks are flattened into one-dimensional vectors, forming a vital input for model fusion and offering a high-dimensional encoded representation of local dependencies within the input sequence.

#### 2.2.2. Transformer

**Figure 7:**
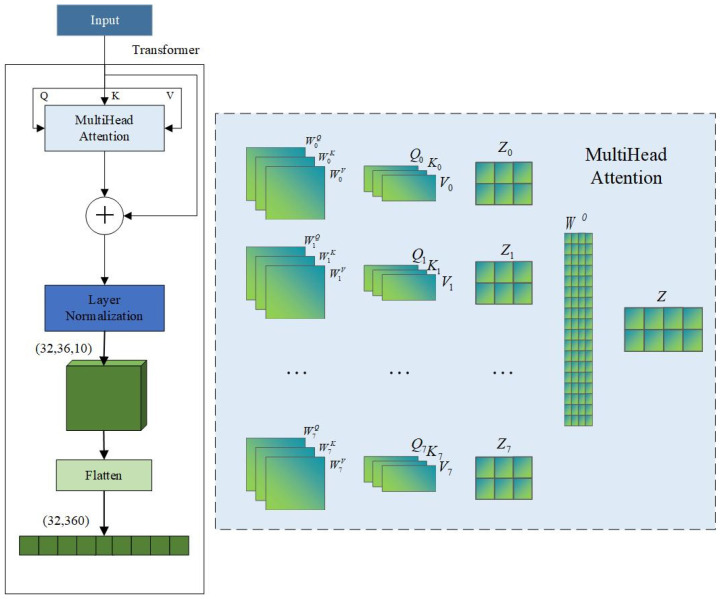
Construction of the Transformer architecture.

The Transformer module captures global dependencies in the input sequence using a multi-head self-attention mechanism [42]. Unlike traditional recurrent neural networks (RNNs), which process sequence data sequentially in time order (see Figure 7), the Transformer computes information exchange between any two sequence positions in parallel. This parallelism avoids gradient vanishing and explosion problems common in RNNs when handling long sequences.

The input features are first linearly transformed across eight heads, corresponding to eight sets of learnable projection matrices ${\left\{W_{i}^{Q},W_{i}^{K},W_{i}^{V}\right\}}_{i=0}^{7}$, generating eight independent attention subspaces. The calculation formula for each attention head is presented in Equation (5).

(5) $$Z_{i}=soft\max(\frac{Q_{i}{K_{y}}^{T}}{\sqrt{d_{k}}})V_{i}$$

where $Q_{i}=XW_{i}^{Q}$, $K_{i}=XW_{i}^{K}$, $V_{i}=XW_{i}^{V}$, X represents the input feature matrix, and $d_{k}$ dk denotes the dimension of the key vectors for each attention head. The scaling factor prevents the inner product from becoming excessively large, which could lead to gradient vanishing. The eight attention heads output attention results ${\left\{Z_{i}\right\}}_{i=0}^{7}$ individually. These results are concatenated into a large tensor and then projected back to the original feature space via a linear transformation matrix $W^{0}$ to produce the overall attention output Z, as shown in Equation (6).

(6) $$Z=Concat(Z_{0},\ldots ,Z_{7})W^{0}$$

To enhance training stability and information retention, the model employs residual connections that add the attention output Z to the original input. Layer Normalization is then applied to standardize each variable sequence’s representation, minimizing discrepancies and reducing noise from non-causal or delayed interactions. Finally, a flattening layer converts the three-dimensional tensor into a one-dimensional vector, allowing concatenation with the TCN branch output and feeding into the subsequent fully connected layer for prediction. This flattened vector captures dependencies across time steps and the global temporal sequence, providing the model with richer global trend information.

#### 2.2.3. Squeeze-and-Excitation (SE)

**Figure 8:**
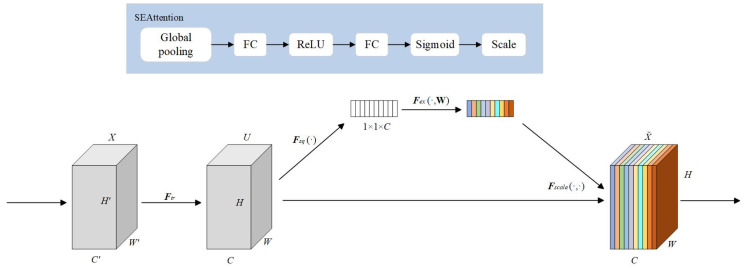
Schematic diagram of Squeeze-and-Excitation.

To improve the model’s capability to distinguish the importance of feature channels, this study incorporates the Squeeze-and-Excitation (SE) channel attention mechanism into the backbone network. Considering the structural characteristics of time series features, the SE-1D module designed for one-dimensional inputs is adopted [43]. The structural diagram and operation flowchart of the SE Attention mechanism are shown in Figure 8.

First, apply global average pooling to the input features to compress each channel along the time dimension and extract the global context information for each channel, as shown in Equation (7):

(7) $$z_{c}=\frac{1}{T}\sum_{t=1}^{T}x_{c,t}$$

Secondly, a two-layer fully connected neural network performs nonlinear mapping on the compressed channel descriptors to generate attention weights for each channel. The first layer reduces dimensionality using the ReLU activation function, decreasing the channel count to C/r. The second layer restores the original channel number and outputs a weight vector $S\in {\left[0,1\right]}^{C}$ via the Sigmoid activation function. These attention weights S are then applied to the original input features by multiplying each channel feature by its corresponding weight, enabling channel-level feature recalibration and enhancing the network’s focus on key features. The process is detailed in Equation (8).

(8) $${\tilde{x}}_{c,t}=s_{c}\cdot x_{c,t}$$

where B denotes the batch size, c represents the number of channels, and t indicates the time steps.

#### 2.2.4. Kalman Filter

To improve the stability and practicality of the model’s predictions, this study applies a one-dimensional Kalman filter after the model output to dynamically smooth the sensible heat temperature prediction sequence. The Kalman filter is a state-space filtering method based on Bayesian estimation theory, assuming that the system state evolves over time and that observed data relate linearly to the system state [44]. By continuously combining historical states with current observations, the filter updates its estimate of the true system state dynamically. This algorithm performs recursive optimal estimation on time series data, effectively removing random noise while preserving trends and avoiding over-smoothing. It is suitable for linear or linearizable systems and is characterized by two fundamental equations: the state transition equation and the observation equation. The state transition equation is presented in Equation (9).

(9) $$x_{k}=Ax_{k-1}+\omega_{k}$$

The observation equation is presented in Equation (10).

(10) $$z_{k}=Hx_{k}+v_{k}$$

where $x_{k}$ denotes the true system state at time *k*, corresponding to the smoothed apparent temperature; $z_{k}$ denotes the observed system value at time k, i.e., the predicted value from the model; A is the state transition matrix; H the observation matrix; $\omega_{k}$ the process noise; and $v_{k}$ the observation noise. The Kalman filter aims to estimate the true state $x_{k}$ by minimizing the mean squared error based on the current observation $z_{k}$.

Compared with other common smoothing techniques, such as simple moving average or exponentially weighted moving average (EWMA), the Kalman filter offers several distinct advantages for this study. First, it dynamically adapts the filtering gain based on the relative uncertainties of the model predictions and observed data, enabling effective smoothing of abrupt fluctuations while preserving long-term trends. Second, it integrates model-predicted values with historical observations, providing a more accurate estimate of the underlying system state for multi-step prediction sequences. Third, its recursive formulation allows real-time updating without storing the entire time series, which is particularly suitable for online environmental monitoring and control in poultry houses. Therefore, the one-dimensional Kalman filter is selected in this study to enhance prediction stability and robustness, ensuring the model outputs are both practical and reliable for intelligent ventilation applications.

### 2.3. Model Evaluation

This study employed 5-fold cross-validation to evaluate model performance, with the dataset divided into training and validation sets in each fold to ensure generalization. During training, mean square error (MSE) was adopted as the loss function to minimize the average squared difference between predicted and actual values. For performance evaluation, three regression metrics were used: mean absolute error (MAE), root mean square error (RMSE), and the coefficient of determination (R^2^). The specific calculation formulas are as shown in Equations (11)–(14).

(11) $$MSE=N^{-1}\sum_{i=1}^{N}{\left({\hat{y}}_{i}-y_{i}\right)}^{2}$$

(12) $$MAE=N^{-1}\sum_{i=1}^{N}\left(|{\hat{y}}_{i}-y_{i}|\right)$$

(13) $$RMSE=\sqrt{N^{-1}\sum_{i=1}^{N}{\left({\hat{y}}_{i}-y_{i}\right)}^{2}}$$

(14) $$R^{2}=1-\frac{\sum_{i=1}^{N}{\left(y_{i}-\hat{y_{i}}\right)}^{2}}{\sum_{i=1}^{N}{\left(y_{i}-\bar{y_{i}}\right)}^{2}}$$

where N denotes the sample size, $y_{i}$ represents the actual output, and $\hat{y_{i}}$ is the predicted value from the model.

## 3. Results

### 3.1. Correlation Analysis

Broiler chickens’ apparent temperature follows a time series pattern with autocorrelation, indicating that previous data influences current changes. Thus, the model must account for both real-time environmental factors and historical data effects. To enhance input parameter effectiveness, reduce redundant features, and lower computational load, this study employed the Pearson correlation coefficient to evaluate correlations among environmental parameters inside and outside the chicken house, control data, and broiler apparent temperature. The Pearson correlation coefficient measures the strength and direction of a linear relationship between two variables, ranging from −1 to 1. Values closer to ±1 indicate stronger correlations. The formula is shown in Equation (15).

(15) $$r=\frac{\sum_{i=1}^{n}\left(x_{i}-\bar{x})(y_{i}-\bar{y}\right)}{\sqrt{\sum_{i=1}^{n}{\left(x_{i}-\bar{x}\right)}^{2}\sum_{i=1}^{n}{\left(y_{i}-\bar{y}\right)}^{2}}}$$

where $x_{i}$ and $y_{i}$ denote the observed values of variables x and y, respectively, while $\bar{x}$ and $\bar{y}$ represent their mean values. The correlation strength corresponding to the Pearson correlation coefficient is defined in Table 3.

**Figure 9:**
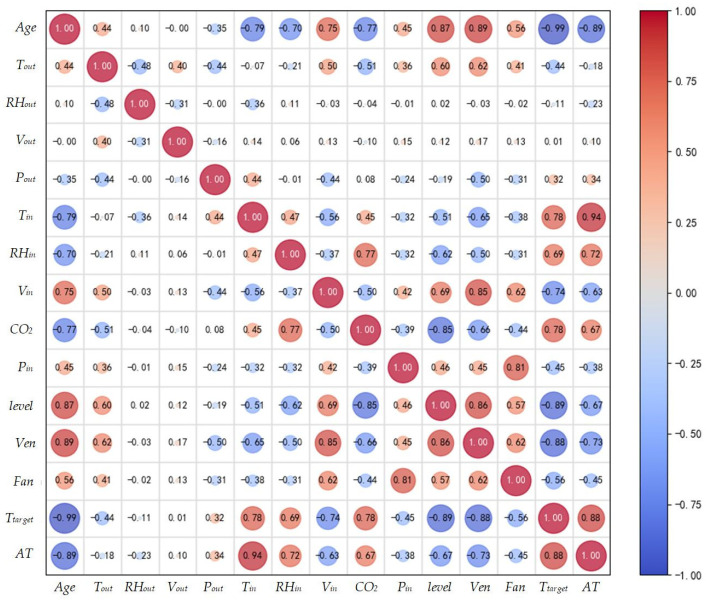
Heatmap for Pearson correlation analysis of experimental data.

As shown in Figure 9, the absolute Pearson correlation coefficients between apparent temperature and outdoor temperature ($T_{out}$), outdoor humidity (${RH}_{out}$), outdoor wind speed ($V_{out}$), atmospheric pressure ($P_{out}$), and negative pressure inside the chicken house ($P_{in}$) are all below 0.4, indicating weak correlations. In contrast, $T_{in}$, ${RH}_{in}$, $V_{in}$, CO_2_, level, Ven, Fan, $T_{target}$ and Age show moderate to strong correlations with broiler apparent temperature and are thus selected as input features. Table 4 lists the parameters included in the broiler apparent temperature prediction model.

**Figure 10:**
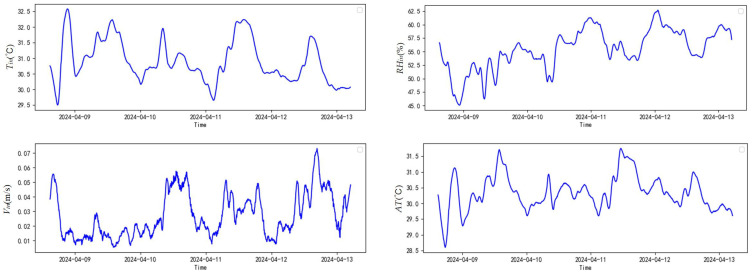
Trends in indoor environmental factors in the henhouse over time.

There is a complex coupling relationship between different environmental factors in the henhouse. Taking the sample of the data set (9–13 April 2024) as an example, Figure 10 shows the trend in indoor environmental factors over time.

Table 5 presents representative raw data from all 12 monitoring sites at several time points, illustrating both temporal fluctuations and spatial heterogeneity within the poultry house. The results clearly show a front–rear gradient: sites 9–12, located near the air inlets, generally exhibited lower temperatures and apparent temperatures due to direct inflow of cooler air, whereas sites 1–4, situated at the rear close to the exhaust fans, tended to be warmer because of reduced airflow and heat accumulation. In addition to this spatial pattern, a pronounced diurnal cycle was observed. During midday (e.g., 9 April, 14:09), the overall indoor temperature and apparent temperature reached peak levels, with the rear region (sites 1–4) exceeding 33 °C, forming localized hot spots, while the front region (sites 9–12) remained 2–3 °C cooler. By contrast, during midnight (e.g., 10 April, 0:39), the indoor environment dropped to its lowest values, and spatial gradients became smaller, though site-dependent differences still persisted. Similar patterns of midday peaks and midnight lows were consistently observed across multiple days. These findings highlight the necessity of multi-point monitoring for capturing both spatial heterogeneity and diurnal variability in the thermal environment.

### 3.2. Algorithm Parameter Setting

The experimental hardware setup includes a 3.6 GHz R7-7745HX CPU, an RTX 4060 GPU, and 32 GB of RAM. The specific hyperparameters are as follows: learning rate of 0.001, Adam optimizer, batch size of 32, up to 200 epochs with early stopping, and input window size of 36 (corresponding to a 3 h horizon at 5 min intervals). The TCN branch consists of four residual blocks with dilation rates of [1, 2, 4, 8], each using 64 filters with a kernel size of 2 and integrated squeeze-and-excitation (SE) attention modules. The Transformer branch employs a single multi-head self-attention layer with 8 heads and key dimension of 2, followed by residual connections and layer normalization. Model outputs are smoothed using a Kalman filter in post-processing. The complete SE-TCN–Transformer–Kalman model required approximately 744.57 s for training, demonstrating computational feasibility for deployment in real-world poultry house environmental control systems. The model was trained and evaluated for short-term forecasting with prediction offsets of t + 1 (5 min), t + 3 (15 min), and t + 6 (30 min).

### 3.3. Comparison of Prediction Performance Among Different Models

To systematically assess the advantages and practical value of our approach, a comparative experiment was conducted without applying Kalman filtering as post-processing. This method was compared against traditional time series models such as LSTM and GRU, as well as advanced Transformer variants like Autoformer and Informer. The comparison aimed to validate the model’s ability to capture multi-scale temporal features, suppress extreme errors, and maintain long-term dependency modeling. Furthermore, the sensitivity of error accumulation across multi-step prediction horizons (5, 15, and 30 min) was analyzed. Results of the comparative study are presented in Table 6.

As the prediction horizon extends from 5 to 30 min, all models exhibit a monotonically increasing trend in prediction errors. The proposed SE-TCN–Transformer model, however, consistently delivers superior performance while maintaining a moderate number of parameters (200, 727), demonstrating a favorable balance between accuracy and computational complexity. For the 5 min horizon, it achieves an MAE of 0.195 °C, RMSE of 0.256 °C, and R^2^ of 0.937, significantly outperforming recurrent models such as LSTM and GRU, as well as Transformer variants including Autoformer and Informer. At 15 and 30 min horizons, the SE-TCN–Transformer maintains low errors (MAE: 0.218 °C and 0.265 °C; RMSE: 0.288 °C and 0.346 °C; R^2^: 0.917 and 0.879). In contrast, other models exhibit larger errors and lower goodness-of-fit. While Informer slightly surpasses Autoformer in R^2^ at 30 min (0.839 vs. 0.818), its overall error metrics remain inferior to SE-TCN–Transformer. Overall, the SE-TCN–Transformer achieves a strong trade-off between predictive accuracy and model complexity, demonstrating practical feasibility for deployment in commercial poultry house environments.

**Figure 11:**
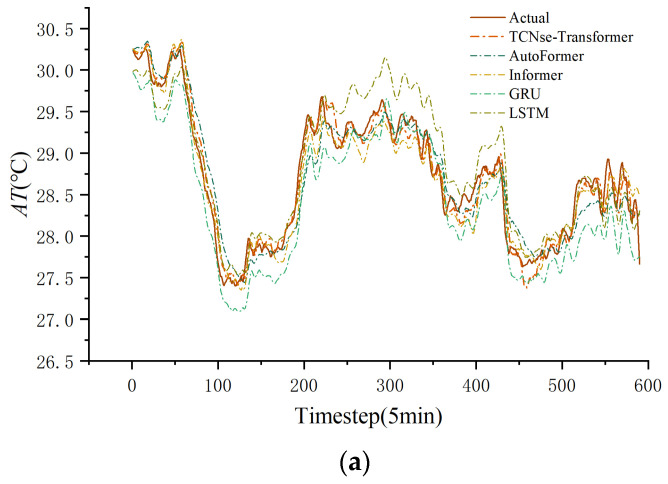

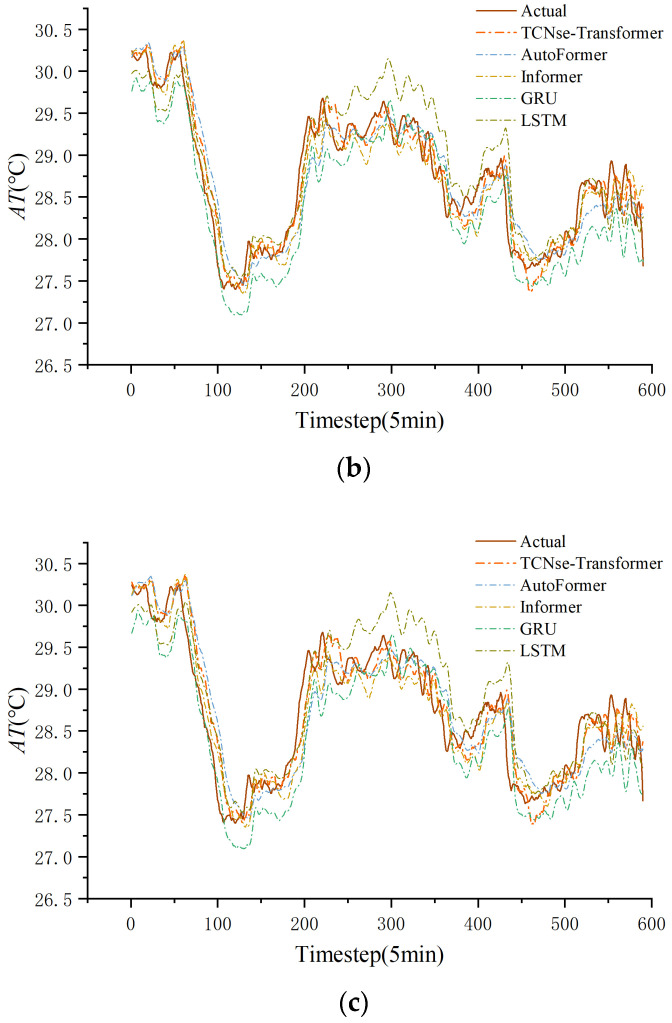
Predicted fitting curves of each model at three prediction steps: (**a**) 5 min; (**b**) 15 min; (**c**) 30 min.

To further validate each model’s fitting performance at different prediction horizons, predicted and actual apparent temperature curves from the test set were plotted at prediction steps t + 1 (5 min), t + 3 (15 min), and t + 6 (30 min), as shown in Figure 11.

As shown in Figure 11a, during the t + 1 (5 min) prediction task, all models closely fit the actual temperature curve, demonstrating strong consistency with overall trends. Among them, the SE-TCN–Transformer model’s predictions closely match the actual values, accurately capturing local fluctuations and exhibiting excellent short-term forecasting ability. Autoformer and Informer closely follow with good fitting results. However, GRU and LSTM show slight deviations at some peaks and valleys, causing minor under- or overestimation. In Figure 11b, with the prediction step length extended to t + 3 (15 min), the models’ responsiveness to local changes declines, increasing prediction errors. Nevertheless, the SE-TCN–Transformer maintains outstanding trend-fitting ability, closely tracking the actual curve across multiple high-frequency fluctuation segments. Overall, Autoformer performs slightly worse than Informer. In contrast, deviations in GRU and LSTM models increase further, showing clear volatility lag and error propagation. For Figure 11c, prediction accuracy of all models significantly decreases in the t + 6 (30 min) task due to the greater challenge of modeling long-term dependencies. Nevertheless, the SE-TCN–Transformer still accurately predicts temperature trends, demonstrating strong robustness and generalization. Autoformer performs relatively steadily in some periods but lacks adequate response to extreme points. Informer’s performance fluctuates, especially with limited predictive power in areas of intense local changes. RNN-based models (GRU and LSTM) show the largest errors at this step, with fitted curves deviating notably from actual values, displaying lag and amplitude compression. Transformer-based models (Autoformer and Informer) demonstrate strong competitiveness in short- and medium-term predictions, whereas traditional RNN models struggle with long-term tasks and need improved long-range dependency modeling. The SE-TCN–Transformer model delivers the best prediction performance and stability across all three prediction horizons, validating its effectiveness and adaptability for modeling both short- and long-term temperature changes.

### 3.4. Ablation Experiment

To evaluate the contribution of each functional module to the multi-step apparent temperature prediction task, this study conducted ablation experiments based on the full SE-TCN–Transformer–Kalman model and quantitatively analyzed each module’s performance using multiple evaluation metrics. The experimental results are presented in Table 7.

The results of the ablation study demonstrate the advantages of the proposed SE-TCN–Transformer architecture in multi-step apparent temperature prediction. Models relying solely on either the TCN or Transformer architecture underperformed across all prediction horizons (t + 1, t + 3, and t + 6) compared to the hybrid model. The base TCN model achieved MAE values of 0.270 °C, 0.296 °C, and 0.331 °C, RMSE values of 0.353 °C, 0.385 °C, and 0.437 °C, and R^2^ scores of 0.874, 0.850, and 0.807 for the 5, 15, and 30 min predictions, respectively, reflecting limited capability in capturing global dependencies, particularly in medium- to long-term forecasts.

Although the Transformer model can capture both short- and long-term dependencies, its short-term prediction performance was inferior in our experiments. In the ablation studies, all modules maintained the same parameter settings as the complete model to isolate the contribution of each module, ensuring that performance differences arise from the module itself rather than hyperparameter variation or data size. Additionally, Transformer models typically require larger datasets to achieve full predictive potential, and the multi-head attention mechanism imposes high computational and memory costs, especially for long sequences. In our tests, the Transformer outperformed TCN in medium- and long-term predictions, achieving an MAE of 0.213 °C, RMSE of 0.280 °C, and R^2^ of 0.921 for the 5 min prediction and R^2^ scores of 0.895 and 0.855 for 15 and 30 min horizons, respectively.

By integrating Transformer with TCN in a dual-branch design, the proposed model effectively addresses these limitations. The TCN branch efficiently extracts local short-term features, reducing reliance on large-scale training data and addressing the Transformer’s shortcomings in short-term prediction. Meanwhile, in this study the Transformer branch operates on relatively short input sequences (time step = 36) and is integrated with the TCN branch only at the feature fusion stage. This design keeps the computational burden of multi-head attention manageable, while still enabling the capture of global dependencies for improved long-sequence representation. This design preserves the model’s ability to capture long-term dependencies while enhancing computational efficiency and practical deployment capabilities.

Incorporating the SE attention module further enhances the model’s ability to identify and emphasize informative channel features. Compared to the base TCN, the SE-TCN module reduced MAE to 0.231 °C, 0.252 °C, and 0.283 °Cand RMSE to 0.305 °C, 0.336 °C, and 0.379 °C and increased R^2^ to 0.906, 0.886, and 0.855 for the 5, 15, and 30 min predictions, respectively, confirming the effectiveness of channel-wise attention.

The full SE-TCN–Transformer hybrid model achieved superior performance, with MAE of 0.195 °C, 0.218 °C, and 0.265 °C, RMSE of 0.256 °C, 0.288 °C, and 0.346 °C, and R^2^ of 0.937, 0.917, and 0.879 for t + 1, t + 3, and t + 6, respectively. Applying a Kalman filter as post-processing further stabilized predictions, suppressing high-frequency noise and improving short-term accuracy. For example, 5 min predictions achieved an MAE of 0.168 °C, RMSE of 0.222 °C, and R^2^ of 0.950, representing improvements of approximately 13.8% and 13.3% compared with the model without Kalman filtering. Similar improvements were observed at 15 and 30 min horizons. Overall, the ablation study confirms that the dual-branch SE-TCN–Transformer with SE attention and Kalman smoothing effectively integrates multi-scale temporal information, balances local and global feature extraction, and achieves high accuracy, robustness, and computational efficiency across multiple prediction steps.

**Figure 12:**
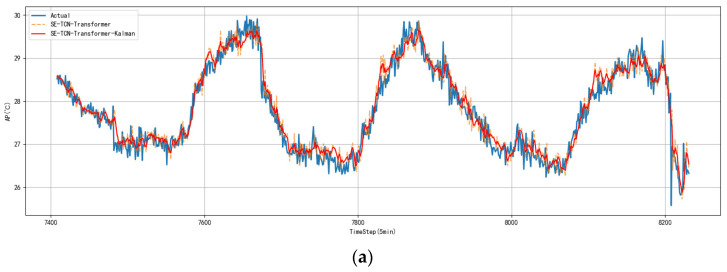

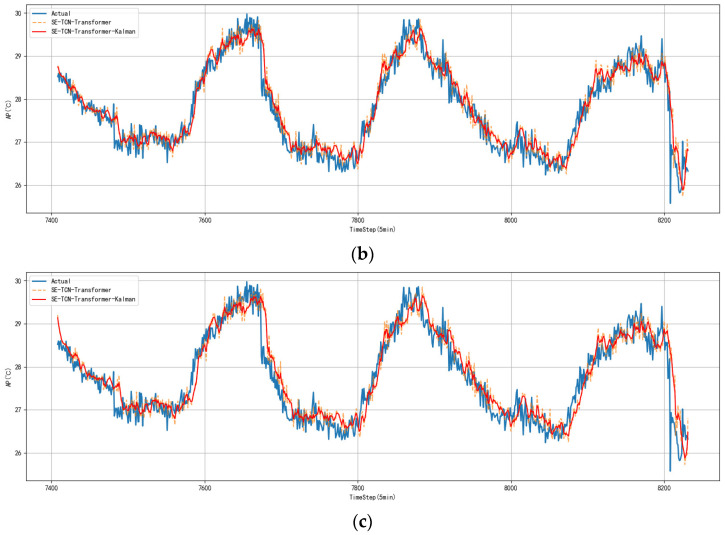
Comparison of actual values and model predictions before and after Kalman smoothing. (**a**) 5 min; (**b**) 15 min; (**c**) 30 min.

To intuitively demonstrate the smoothing effect of Kalman filtering on the model’s multi-step predictions, the predicted curve from the SE-TCN–Transformer model, the Kalman-filtered curve, and the actual apparent temperature curve from the test set are plotted together in the same coordinate system, and the results are shown in Figure 12.

In the test set, prediction curves at all prediction steps accurately captured the double-peak pattern of daytime warming and nighttime cooling. However, unsmoothed predictions displayed noticeable jagged edges during periods of rapid fluctuation. After Kalman filtering, the curves closely followed the actual trends and effectively suppressed instantaneous peaks, clearly demonstrating the Kalman module’s important role in enhancing the stability of dynamic apparent temperature predictions. As shown in Figure 12a, the model achieves exceptionally high accuracy in 5 min ultra-short-term predictions, with overall trends and phases highly consistent, reflecting strong dynamic response and robustness. In Figure 12b, the 15 min predictions remain accurate, successfully capturing the main patterns of the twice-daily temperature fluctuations, including both the midday peak and early-morning trough. Some slight lag occurs during rapid changes, particularly during nighttime rapid cooling, primarily due to mid-term prediction sensitivity to diminishing input information. After Kalman smoothing, the R^2^ improved from 0.917 to 0.931, confirming that the filter enhances model performance in dynamic, non-stationary regions. Despite some degradation compared to the 5 min results, the model maintains good adaptability. Figure 12c shows that in the 30 min prediction scenario, the model still fits overall trends well and captures periodic changes like midday warming and nighttime cooling. However, compared to shorter-term predictions, local detail lag and extreme value deviations are more pronounced, especially during rapid warming and cooling phases. The R^2^ decreases to 0.879, indicating that the model preserves trend recognition for long-term forecasts but responds more slowly and with increased errors during extreme fluctuations. After Kalman filtering, R^2^ improves to 0.895, showing that the filter effectively removes local noise and enhances curve interpretability.

### 3.5. Model Error Analysis

**Figure 13:**
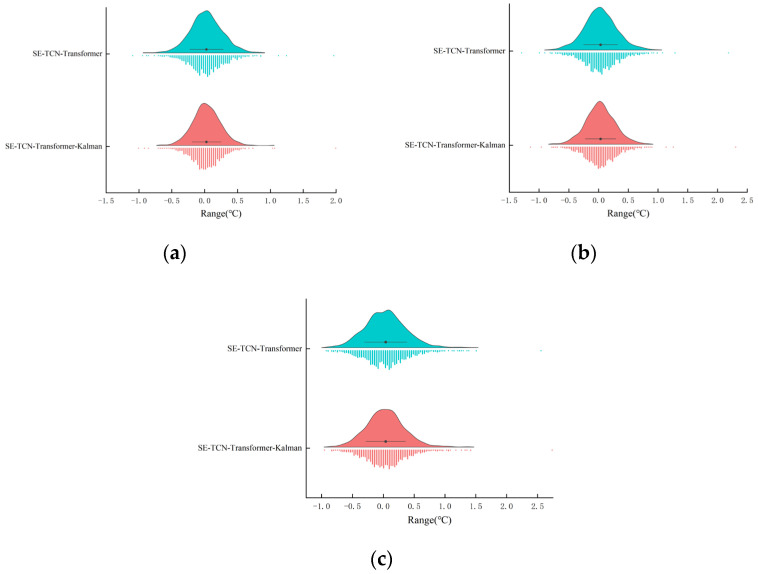
Error distribution of model predictions before and after Kalman smoothing. (**a**) 5 min; (**b**) 15 min; (**c**) 30 min.

To further assess predictive performance, this study compares error statistics and distributions of the SE-TCN–Transformer model and its Kalman filter-enhanced variant at three prediction steps (t + 1, t + 3, and t + 6), as summarized in Table 8 and visualized in Figure 13.

Overall, both models’ errors are centered near zero, indicating no systematic bias. Incorporating the Kalman filter leads to markedly improved stability, reflected by reductions in the interquartile range (14.6%, 6.7%, and 11.1% for the three steps) and fewer outliers in short- and medium-term forecasts (from 30 to 25 at t + 1 and from 35 to 28 at t + 3). The error distributions also become more concentrated around 0 °C, with steeper and more symmetric density curves, demonstrating suppressed error fluctuations and enhanced robustness. For the 5 and 15 min forecasts, the Kalman-fused model shows narrower error ranges and fewer extreme deviations compared with the baseline. At 30 min, both models exhibit wider spreads and a modest increase in extreme errors, suggesting a trade-off between smoothing and sensitivity to abrupt changes in long-term predictions. Taken together, the statistical and visual evidence confirms that Kalman filtering significantly improves accuracy and stability in short- and medium-term forecasting, while maintaining relatively robust performance under longer horizons.

## 4. Discussion

The thermal environment in poultry houses is influenced by multiple factors—temperature, humidity, and wind speed—forming a complex and highly nonlinear system that challenges modeling and control [45]. Heat stress is well-documented to significantly inhibit broiler production performance, making accurate prediction of apparent temperature at various time points essential for intelligent ventilation control. Apparent temperature serves not only as a fundamental parameter for heat stress index calculation but also as a direct indicator of chicken comfort and production performance. Therefore, developing a high-precision apparent temperature prediction model is critical for optimizing production environment management and feeding decisions. This study proposes a multi-step prediction model for broiler apparent temperature that integrates the SE-TCN–Transformer architecture with a Kalman filter, using apparent temperature (AT) as the direct prediction target. This approach avoids the complexity and error accumulation associated with separate modeling and combined prediction of multiple parameters like temperature, humidity, and wind speed in traditional methods. As a comprehensive thermal indicator, apparent temperature captures the interactive effects of temperature, humidity, and wind, providing an intuitive reflection of chickens’ thermal comfort with strong physiological relevance and practical engineering value. Its averaging and functional properties also suppress sensor instantaneous drift and spatial heterogeneity interference, reducing dependence on input feature decoupling and enhancing overall prediction robustness. Architecturally, the TCN module employs causal convolution to preserve temporal order and dilated convolution to expand the receptive field, effectively capturing short-term local fluctuations. The SE module incorporates a channel attention mechanism to amplify key feature pathways. Meanwhile, the Transformer module models long-range dependencies via a multi-head self-attention mechanism, uncovering periodicity and trend changes in apparent temperature. The synergy of these components strengthens the model’s ability to extract both local and global temporal features. Furthermore, the Kalman filter enhances dynamic smoothness by fusing model predictions with historical state estimates, effectively suppressing sudden fluctuations such as abrupt wind speed changes, thereby improving the model’s adaptability and robustness.

To comprehensively assess the model’s prediction accuracy and stability, this study compared the performance of the SE-TCN–Transformer model and its Kalman filter-enhanced variant (SE-TCN–Transformer–Kalman) across multi-step prediction tasks. In short-term predictions (t + 1, 5 min), the Kalman-enhanced model achieved the lowest MAE (0.168 °C) and RMSE (0.222 °C), significantly improving over the unfiltered version (MAE of 0.195 °C and RMSE of 0.256 °C). Furthermore, the coefficient of determination (R^2^) increased from 0.937 to 0.950, reflecting enhanced accuracy and fitting quality. In mid-term (t + 3, 15 min) and long-term (t + 6, 30 min) predictions, the Kalman filter continued to deliver notable improvements, with R^2^ rising by approximately 1.4 and 1.6 percentage points, respectively, confirming its effectiveness in stabilizing long-term multi-step prediction sequences.

From the perspective of error distribution, the median residuals of both models are close to zero, indicating no overall systematic bias. However, the Kalman filter significantly reduces error dispersion. For instance, in the 30 min forecast, the interquartile range (IQR) decreased from 0.424 to 0.377, showing that forecast errors became more concentrated and volatility decreased. Notably, the Q1-to-Q3 interval shifted left and compressed, resulting in a more stable and reliable forecast interval. Although the maximum error slightly increased after applying the filter (rising from 2.548 to 2.722 at t + 6), the overall error distribution became more symmetrical and concentrated. This indicates that while Kalman filtering cannot eliminate all extreme values, it effectively buffers most noise-induced disturbances, enhancing the model’s robustness and practical applicability in real livestock farming environments.

Although classical time series methods such as ARIMA could theoretically be applied to predict the thermal environment in poultry houses and capture trends and seasonality, the actual environment is influenced by multiple interacting factors, including temperature, humidity, wind speed, and CO_2_ concentration, exhibiting strong nonlinearity and rapid fluctuations. ARIMA assumes linear relationships and has limited capacity to model multivariate interactions, making it difficult to capture both complex nonlinear dependencies and long- and short-term temporal correlations, which limits its suitability for high-precision, multivariate, multi-step forecasting tasks [46,47].

On the other hand, incorporating physical models such as hydrodynamic climate processes can enhance the interpretability of predictions. However, high-fidelity physical modeling requires detailed parameter measurements (e.g., airflow distribution and heat and moisture transfer coefficients) and extensive experimental data, and it entails high computational complexity, which limits its applicability for real-time multi-step forecasting and rapid ventilation control. In practice, such approaches often struggle to balance predictive accuracy and real-time performance [48,49].

Compared with the existing research, this work adopts a data-driven deep learning method, integrates TCN and Transformer architectures to predict the thermal environment in livestock and poultry houses, and directly captures the environmental dynamics through multi-sensor data, which not only ensures the prediction accuracy, but also facilitates real-time application.

Recent advances such as SCINet, Informer, and Autoformer have demonstrated impressive performance in generic long-sequence forecasting tasks. For example, SCINet has been employed in multi-scale power load prediction [50], Informer has been applied to hydrological flood discharge forecasting [51], and Autoformer has achieved strong results in electricity load prediction [52]. However, these architectures are typically optimized for large-scale datasets and generic long-sequence tasks, which may limit their effectiveness in more specialized domains. In contrast, our study addresses apparent temperature prediction in broiler houses, where data are relatively constrained and strongly influenced by multi-sensor fusion from a complex indoor environment. Under such conditions, the advantages of SCINet, Informer, or Autoformer are less pronounced. The proposed SE-TCN–Transformer dual-branch model provides a tailored balance: the SE-TCN branch efficiently captures short-term local variations, while the Transformer branch models long-term dependencies within manageable computational costs. This design yields robust prediction accuracy and stability under real-world data limitations, making the model particularly suitable for practical deployment in intelligent ventilation management and heat stress early-warning systems in poultry farming. It employs the SE attention mechanism to enhance the model’s focus on representative local features and incorporates Kalman filtering in the post-processing stage to improve the numerical accuracy of apparent temperature predictions. Moreover, this approach enhances the stability and robustness of the prediction sequence, especially in multi-step forecasting scenarios. Serving as a dynamic smoothing supplement, the Kalman filter complements the SE-TCN–Transformer model, offering a solution for thermal environment modeling in livestock facilities that aligns more closely with practical control requirements. This advancement supports future applications such as intelligent ventilation and heat stress warning systems.

Although the model’s performance has improved significantly, several limitations persist. First, data collection currently relies on averaged values from fixed-point sensors, potentially overlooking local thermal stress due to spatial heterogeneity. Second, the training dataset does not encompass extreme environmental conditions or equipment malfunctions. Future work could address this by employing adversarial training and transfer learning to enhance generalization. Third, the model has yet to be integrated into a closed-loop ventilation control system. Seasonal variations also play an important role in the thermal environment of poultry houses, influencing temperature, humidity, and wind patterns, and consequently affecting apparent temperature and broiler comfort. For instance, summer months often present higher ambient temperatures and humidity, potentially exacerbating heat stress, while winter months may introduce cold stress and require different ventilation strategies. Although the current study focuses on a single season, incorporating seasonal variability into model training could further enhance its generalization and robustness across different climatic conditions. Future research should explore combining online learning with reinforcement learning, perform multi-period validation, and leverage cross-seasonal and cross-annual datasets to evaluate the model’s predictive performance under diverse environmental conditions, enabling real-time dynamic environmental regulation. This approach will facilitate assessment of both short-term and long-term prediction accuracy, as well as the model’s stability and adaptability under seasonal fluctuations. Moreover, since the current apparent temperature calculation relies on empirical formulas, future studies could integrate infrared imaging to monitor broiler behavioral and physiological indicators—such as mouth opening rate, flocking behavior, and heart rate—to develop physiologically driven heat stress modeling and prediction systems, thereby enhancing the biological relevance and practical applicability of the model.

## 5. Conclusions

This study addresses challenges in broiler farming environmental control delays and the difficulty of representing chicken thermal comfort using a single parameter by proposing a multi-step apparent temperature prediction model that integrates the SE-TCN–Transformer architecture with a Kalman filter. The TCN module efficiently extracts short-term local features, while the Transformer module captures long-range dependencies and periodic trends. The SE attention mechanism enhances key feature representation, and the Kalman filter improves prediction smoothness and robustness by mitigating sudden fluctuations and noise in post-processing. Experimental results demonstrate that the model outperforms traditional RNN-based and Transformer variants in 5, 15, and 30 min prediction tasks. It also shows significant advantages in error distribution stability, outlier suppression, and prediction interval convergence, confirming its applicability and robustness in dynamic, complex environments. This model provides a high-precision tool for intelligent regulation of poultry farming environments, aiding in optimizing ventilation strategies, reducing heat stress risk, and improving animal welfare and economic benefits. Future research will focus on multi-scale spatial data fusion, model lightweight deployment, and deeper integration with automated control systems, including exploring closed-loop ventilation control. Furthermore, incorporating behavioral and physiological signals—such as mouth-opening rate, clustering degree, and body surface temperature—will help develop a more biologically driven heat stress prediction system, enhancing adaptability and performance in practical production.

## Figures and Tables

**Table 1:** Relevant parameters of environmental sensors.

Sensor Index	Unit	Range	Accuracy
Temperature	°C	−20~+50 °C	±0.2 °C
Humidity	%	0~100% RH	±2% RH
Wind speed	m/s	0.1~5 m/s	±(0.2 m/s ± 0.02 × v)
Carbon dioxide	ppm	300~5000 ppm	±50 ppm
Negative Pressure	Pa	0~20,000 Pa	±5 Pa
Atmospheric Pressure	Pa	9000~110,000 Pa	±100 Pa

**Table 3:** Definition of correlation degree of Pearson correlation coefficient.

Correlation Level	Extremely Relevant	Strong Relevant	Medium Relevant	Weak Relevant	Extremely Weak Relevant	Irrelevant
Coefficient	0.8 < |r| ≤ 1.0	0.6 < |r| ≤ 0.8	0.4 < |r| ≤ 0.6	0.2 < |r| ≤ 0.4	0 < |r| ≤ 0.2	0

**Table 4:** Parameter description and unit.

Parameter	Description	Unit
*Age*	Age of broiler chickens	Day
$T_{in}$	Indoor temperature	°C
${RH}_{in}$	Indoor humidity	%
$V_{in}$	Indoor wind speed	m/s
*CO*_2_	CO_2_ concentration in the house	ppm
*level*	Grades of ventilation	level
*Ven*	Ventilation rate	m^3^/h
*Fan*	Number of fans	-
$T_{target}$	Target temperature	°C
*AT*	Apparent temperature	°C

**Table 5:** Representative raw data of 12 monitoring points at several time points.

Time	Parameter	Monitoring Sites											
		1	2	3	4	5	6	7	8	9	10	11	12
9 April 2024 14:09	*T_in_* (°C)	32.60	32.90	32.80	32.6	32.30	33.20	33.20	32.40	31.20	31.50	31.30	30.80
	*RH_in_* (%)	60.80	60.20	65.00	61.60	59.20	48.50	48.20	57.10	52.00	51.40	50.90	50.70
	*AT* (°C)	32.68	32.92	33.30	32.76	32.22	32.05	32.02	32.11	30.40	30.64	30.39	29.87
10 April 2024 0:39	*T_in_* (°C)	29.60	29.80	29.80	29.50	30.40	31.20	31.00	30.56	30.00	30.00	30.00	29.50
	*RH_in_* (%)	63.70	63.10	65.30	62.90	53.10	48.20	47.00	52.95	50.10	47.80	47.30	48.80
	*AT* (°C)	29.32	30.11	30.33	29.79	29.71	30.02	29.05	29.86	29.01	28.78	28.73	28.38
10 April 2024 14:22	*T_in_* (°C)	31.70	32.20	32.20	31.70	31.20	32.00	32.00	31.64	30.00	30.20	30.10	29.50
	*RH_in_* (%)	60.20	55.70	60.20	57.30	53.50	50.80	51.30	53.03	59.00	58.30	57.40	57.20
	*AT* (°C)	31.72	31.77	32.22	31.43	30.55	31.08	31.13	30.94	29.90	30.03	29.84	29.22
11 April 2024 0:50	*T_in_* (°C)	30.40	30.50	30.60	30.40	30.30	31.10	31.00	30.60	29.20	29.30	29.20	28.70
	*RH_in_* (%)	66.60	65.90	68.00	66.60	62.00	52.50	53.60	61.20	57.80	55.70	56.80	54.80
	*AT* (°C)	31.06	31.09	31.04	31.06	30.20	30.35	30.36	30.72	28.98	28.87	28.88	28.18
11 April 2024 14:06	*T_in_* (°C)	32.50	33.00	33.10	32.60	32.20	32.90	33.00	32.40	31.40	31.40	31.70	31.00
	*RH_in_* (%)	60.10	54.80	51.00	51.90	52.00	49.00	49.00	51.20	58.30	58.60	54.70	56.50
	*AT* (°C)	32.31	32.18	32.20	30.79	31.40	31.80	31.90	31.52	31.23	31.26	31.17	30.65

**Table 6:** Comparison of model performance at different prediction steps.

Model	Total Number of Parameters	Metric	Prediction Step		
			t + 1 (5 min)	t + 3 (15 min)	t + 6 (30 min)
LSTM	94,949	MAE (°C)	0.312	0.331	0.360
		RMSE (°C)	0.394	0.424	0.473
		R^2^	0.843	0.819	0.774
GRU	72,869	MAE (°C)	0.285	0.300	0.324
		RMSE (°C)	0.356	0.382	0.425
		R^2^	0.872	0.853	0.818
Autoformer	84,017	MAE (°C)	0.250	0.281	0.326
		RMSE (°C)	0.326	0.367	0.425
		R^2^	0.893	0.864	0.818
Informer	256,133	MAE (°C)	0.258	0.276	0.304
		RMSE (°C)	0.332	0.359	0.399
		R^2^	0.889	0.870	0.839
SE-TCN- Transformer	200,727	MAE (°C)	0.195	0.218	0.265
		RMSE (°C)	0.256	0.288	0.346
		R^2^	0.937	0.917	0.879

**Table 7:** Ablation results for model apparent temperature predictions with different prediction steps.

Model	Metric	Prediction Step		
		t + 1 (5 min)	t + 3 (15 min)	t + 6 (30 min)
TCN	MAE (°C)	0.270	0.296	0.331
	RMSE (°C)	0.353	0.385	0.437
	R^2^	0.874	0.850	0.807
Transformer	MAE (°C)	0.213	0.247	0.290
	RMSE (°C)	0.280	0.322	0.379
	R^2^	0.921	0.895	0.855
SE-TCN	MAE (°C)	0.231	0.252	0.283
	RMSE (°C)	0.305	0.336	0.379
	R^2^	0.906	0.886	0.855
SE-TCN–Transformer	MAE (°C)	0.195	0.218	0.265
	RMSE (°C)	0.256	0.288	0.346
	R^2^	0.937	0.917	0.879
SE-TCN– Transformer–Kalman	MAE (°C)	0.168	0.197	0.244
	RMSE (°C)	0.222	0.261	0.328
	R^2^	0.950	0.931	0.895

**Table 8:** Error analysis of model prediction before and after Kalman smoothing.

Error Statistics	SE-TCN–Transformer			SE-TCN–Transformer–Kalman		
	t + 1	t + 3	t + 6	t + 1	t + 3	t + 6
Min	−1.080	−1.286	−0.924	−1.009	−1.131	−0.955
Q1	−0.131	−0.138	−0.184	−0.105	−0.129	−0.162
Median	0.023	0.028	0.035	0.022	0.024	0.027
Q3	0.184	0.205	0.240	0.164	0.191	0.214
Max	1.963	2.199	2.548	1.985	2.300	2.722
IQR	0.315	0.343	0.424	0.269	0.320	0.377
Outler Count	30	35	35	25	28	37

## Data Availability

The data provided in this study are available upon request from the corresponding author.

## References

[1] FAO (Global Perspective Studies Unit) (2006). World Agriculture Towards 2030/2050.

[2] Mazunga F., Mzikamwi T., Mazunga G., Mashasha M., Mazheke V. (2023). IoT based remote poultry monitoring systems for improving food security and nutrition: Recent trends and issues. J. Agric. Sci. Technol..

[3] Zhang C., Zhao X.H., Yang L., Chen X.Y., Jiang R.S., Jin S.H., Geng Z.Y. (2017). Resveratrol alleviates heat stress-induced impairment of intestinal morphology, microflora, and barrier integrity in broilers. Poult. Sci..

[4] Quinteiro-Filho W.M., Rodrigues M.V., Ribeiro A., Ferraz-de-Paula V., Pinheiro M.L., Sá L.R.M., Ferreira A.J.P., Palermo-Neto J. (2012). Acute heat stress impairs performance parameters and induces mild intestinal enteritis in broiler chickens: Role of acute hypothalamic-pituitary-adrenal axis activation. J. Anim. Sci..

[5] Sohail M.U., Hume M.E., Byrd J.A., Nisbet D.J., Ijaz A., Sohail A., Shabbir M.Z., Rehman H. (2012). Effect of supplementation of prebiotic mannan-oligosaccharides and probiotic mixture on growth performance of broilers subjected to chronic heat stress. Poult. Sci..

[6] Rodrigues M., Garcia Neto M., Perri S., Sandre D., Faria M., Oliveira P., Pinto M., Cassiano R. (2019). Techniques to minimize the effects of acute heat stress or chronic in broilers. Braz. J. Poult. Sci..

[7] de Souza L.F.A., Espinha L.P., de Almeida E.A., Lunedo R., Furlan R.L., Macari M. (2016). How heat stress (continuous or cyclical) interferes with nutrient digestibility, energy and nitrogen balances and performance in broilers. Livest. Sci..

[8] Astill J., Dara R.A., Fraser E.D., Roberts B., Sharif S. (2020). Smart poultry management: Smart sensors, big data, and the internet of things. Comput. Electron. Agric..

[9] Lin H., Zhang H.F., Du R., Gu X.H., Zhang Z.Y., Buyse J., Decuypere E. (2005). Thermoregulation responses of broiler chickens to humidity at different ambient temperatures. II. Four weeks of age. Poult. Sci..

[10] Dozier W.A., Lott B.D., Branton S.L. (2005). Growth responses of male broilers subjected to increasing air velocities at high ambient temperatures and a high dew point. Poult. Sci..

[11] Akter S., Cheng B., West D., Liu Y., Qian Y., Zou X., Classen J., Cordova H., Oviedo E., Wang-Li L. (2022). Impacts of Air Velocity Treatments under Summer Condition: Part I—Heavy Broiler’s Surface Temperature Response. Animals.

[12] Nascimento S.T., da Silva I.J.O., Maia A.S.C., de Castro A.C., Vieira F.M.C. (2014). Mean surface temperature prediction models for broiler chickens—A study of sensible heat flow. Int. J. Biometeorol..

[13] Yahav S. (2009). Alleviating heat stress in domestic fowl: Different strategies. World’s Poult. Sci. J..

[14] Tao X., Xin H. (2003). Acute synergistic effects of air temperature, humidity, and velocity on homeostasis of market-size broilers. Trans. ASAE.

[15] García R., Aguilar J., Toro M., Pinto A., Rodríguez P. (2020). A systematic literature review on the use of machine learning in precision livestock farming. Comput. Electron. Agric..

[16] Berckmans D. (2017). General introduction to precision livestock farming. Anim. Front..

[17] Wu Z., Yang J., Zhang H., Fang C. (2025). Enhanced Methodology and Experimental Research for Caged Chicken Counting Based on YOLOv8. Animals.

[18] Trentin A., Talamini D.J.D., Coldebella A., Pedroso A.C., Gomes T.M.A. (2025). Technical and economic performance favours fully automated climate control broiler housing. Br. Poult. Sci..

[19] Gautam K.R., Zhang G., Landwehr N., Adolphs J. (2021). Machine learning for improvement of thermal conditions inside a hybrid ventilated animal building. Comput. Electron. Agric..

[20] Ali M., Imran M., Baig M.S., Shah A., Ullah S.S., Alroobaea R., Iqbal J. (2024). Intelligent control shed poultry farm system incorporating with machine learning. IEEE Access.

[21] Hu C., Li L., Jia Y., Xie Z., Yu Y., Huo L. (2024). CFD Investigation on Combined Ventilation System for Multilayer-Caged-Laying Hen Houses. Animals.

[22] Li L., Li M., Yu Y., Jia Y., Qian Z., Xie Z. (2024). Modeling and Regulation of Dynamic Temperature for Layer Houses Under Combined Positive- and Negative-Pressure Ventilation. Animals.

[23] Costantino A., Fabrizio E., Ghiggini A., Bariani M. (2018). Climate control in broiler houses: A thermal model for the calculation of the energy use and indoor environmental conditions. Energy Build..

[24] Norton T., Sun D.-W., Grant J., Fallon R., Dodd V. (2007). Applications of computational fluid dynamics (CFD) in the modelling and design of ventilation systems in the agricultural industry: A review. Bioresour. Technol..

[25] Bjerg B., Cascone G., Lee I.-B., Bartzanas T., Norton T., Hong S.-W., Seo I.-H., Banhazi T., Liberati P., Marucci A. (2013). Modelling of ammonia emissions from naturally ventilated livestock buildings. Part 3: CFD modelling. Biosyst. Eng..

[26] Selle M., Spieß F., Visscher C., Rautenschlein S., Jung A., Auerbach M., Hartung J., Sürie C., Distl O. (2023). Real-Time Monitoring of Animals and Environment in Broiler Precision Farming—How Robust Is the Data Quality?. Sustainability.

[27] Neethirajan S. (2020). The role of sensors, big data and machine learning in modern animal farming. Sens. Bio-Sens. Res..

[28] Martinez A.A.G., Nääs I.d.A., de Carvalho-Curi T.M.R., Abe J.M., Lima N.D.d.S. (2021). A Heuristic and data mining model for predicting broiler house environment suitability. Animals.

[29] Gao L., Er M., Li L., Wen P., Jia Y., Huo L. (2022). Microclimate environment model construction and control strategy of enclosed laying brooder house. Poult. Sci..

[30] Chen X., Yang L., Xue H., Li L., Yu Y. (2023). A Machine Learning Model Based on GRU and LSTM to Predict the Environmental Parameters in a Layer House, Taking CO_2_ Concentration as an Example. Sensors.

[31] Rodriguez M.R., Besteiro R., Ortega J.A., Fernandez M.D., Arango T. (2022). Evolution and Neural Network Prediction of CO_2_ Emissions in Weaned Piglet Farms. Sensors.

[32] Küçüktopçu E., Cemek B., Simsek H. (2024). Machine Learning and Wavelet Transform: A Hybrid Approach to Predicting Ammonia Levels in Poultry Farms. Animals.

[33] Xu Y., Teng G., Zhou Z. (2024). Short-Term Prediction Method for Gas Concentration in Poultry Houses Under Different Feeding Patterns. Agriculture.

[34] Guo Z., Yin Z., Lyu Y., Wang Y., Chen S., Li Y., Zhang W., Gao P. (2024). Research on Indoor Environment Prediction of Pig House Based on OTDBO–TCN–GRU Algorithm. Animals.

[35] Bai S., Kolter J.Z., Koltun V. (2018). An empirical evaluation of generic convolutional and recurrent networks for sequence modeling. arXiv.

[36] Ji H., Teng G. (2025). Multistep prediction of temperature and humidity in poultry houses based on the GFF-transformer model. Front. Agric. Sci. Eng..

[37] Wu H., Xu J., Wang J., Long M. (2021). Autoformer: Decomposition transformers with auto-correlation for long-term series forecasting. Adv. Neural Inf. Process. Syst..

[38] Zhou H., Zhang S., Peng J., Zhang S., Li J., Xiong H., Zhang W. (2021). Informer: Beyond Efficient Transformer for Long Sequence Time-Series Forecasting. Proc. AAAI Conf. Artif. Intell..

[39] Liu M., Zeng A., Chen M., Xu Z., Lai Q., Ma L., Xu Q. (2022). Scinet: Time series modeling and forecasting with sample convolution and interaction. Adv. Neural Inf. Process. Syst..

[40] Fournel S., Rousseau A.N., Laberge B. (2017). Rethinking environment control strategy of confined animal housing systems through precision livestock farming. Biosyst. Eng..

[41] Fan J., Zhang K., Huang Y., Zhu Y., Chen B. (2023). Parallel spatio-temporal attention-based TCN for multivariate time series prediction. Neural Comput. Appl..

[42] Vaswani A., Shazeer N., Parmar N., Uszkoreit J., Jones L., Gomez A.N., Kaiser Ł., Polosukhin I. Attention is all you need. Proceedings of the Neural Information Processing Systems 30 (NIPS 2017).

[43] Hu J., Li S., Gang S. Squeeze-and-excitation networks. Proceedings of the IEEE Conference on Computer Vision and Pattern Recognition.

[44] Särkkä S., Lennart S. (2023). Bayesian Filtering and Smoothing.

[45] Qi F., Zhao X., Shi Z., Li H., Zhao W. (2023). Environmental Factor Detection and Analysis Technologies in Livestock and Poultry Houses: A Review. Agriculture.

[46] Aria S.S., Iranmanesh S.H., Hassani H. (2024). Optimizing Multivariate Time Series Forecasting with Data Augmentation. J. Risk Financ. Manag..

[47] Long L., Liu Q., Peng H., Wang J., Yang Q. (2022). Multivariate time series forecasting method based on nonlinear spiking neural P systems and non-subsampled shearlet transform. Neural Netw..

[48] Küçüktopcu E., Cemek B., Simsek H., Ni J.-Q. (2022). Computational Fluid Dynamics Modeling of a Broiler House Microclimate in Summer and Winter. Animals.

[49] Choi L.-Y., Daniel K.F., Lee S.-Y., Lee C.-R., Park J.-Y., Park J., Hong S.-W. (2024). CFD Simulation of Dynamic Temperature Variations Induced by Tunnel Ventilation in a Broiler House. Animals.

[50] Zhang X., Wang Y., Liu Z., Chen H. (2025). SCINet applied in multi-scale long-sequence power load forecasting. Electronics.

[51] Li J., Huang Y., Zhou M. (2024). Informer-based model for long-term hydrological forecasting of reservoir flood discharge. Water.

[52] Tang L., Zhang Z., Chen J., Xu L., Zhong J., Yuan P. (2023). Research on Autoformer-based electricity load forecasting and analysis. J. East China Norm. Univ. (Nat. Sci.).

